# Augmented Lagrange Based on Modified Covariance Matching Criterion Method for DOA Estimation in Compressed Sensing

**DOI:** 10.1155/2014/241469

**Published:** 2014-02-11

**Authors:** Weijian Si, Xinggen Qu, Lutao Liu

**Affiliations:** Department of Information and Communication Engineering, Harbin Engineering University, Harbin 150001, China

## Abstract

A novel direction of arrival (DOA) estimation method in compressed sensing (CS) is presented, in which DOA estimation is considered as the joint sparse recovery from multiple measurement vectors (MMV). The proposed method is obtained by minimizing the modified-based covariance matching criterion, which is acquired by adding penalties according to the regularization method. This minimization problem is shown to be a semidefinite program (SDP) and transformed into a constrained quadratic programming problem for reducing computational complexity which can be solved by the augmented Lagrange method. The proposed method can significantly improve the performance especially in the scenarios with low signal to noise ratio (SNR), small number of snapshots, and closely spaced correlated sources. In addition, the Cramér-Rao bound (CRB) of the proposed method is developed and the performance guarantee is given according to a version of the restricted isometry property (RIP). The effectiveness and satisfactory performance of the proposed method are illustrated by simulation results.

## 1. Introduction

Direction of arrival (DOA) estimation of multiple narrow-band sources has been an interesting research topic in array signal processing. Its applications span many fields including radar, communication systems, and medical imaging [1, 2]. Many effective algorithms are proposed for DOA estimation, which are mainly classified into three categories. The beamforming algorithms such as MVDR [3] and MEM [4] obtain a nonparametric spatial spectrum by optimizing the filter weights. The subspace algorithms such as MUSIC [5] and ESPRIT [6] and their derivatives [7, 8] exploit the orthogonality of signal subspace and noise subspace for DOA estimation. The subspace fitting algorithms including Maximum Likelihood (ML) [9] and Weighted Subspace Fitting (WSF) [10] solve a multidimensional nonlinear optimization problem to obtain a precise estimation, but a good initialization is required to ensure global convergence and computational complexity is very high. All these algorithms focus on two important issues: resolution (i.e., the ability to correctly resolve two closely spaced sources) and precision (i.e., the deviation from true DOA) which are considered to be the theoretical bases of evaluating certain algorithms. Many high resolution methods suffer from serious performance degradation in the scenarios with low SNR, small number of snapshots, and closely spaced corrected sources. More recently, many research applications involving compressed sensing (CS) [11], especially DOA estimation [12, 13], have become more and more popular in the signal processing. Moreover, the restricted isometry property (RIP) based on the perfect theoretical framework given with modern probability theory by Donoho [11] and Candés et al. [14] provides the performance guarantee in CS. Hence, in this paper, DOA estimation is posed as a joint sparse recovery problem where we recover jointly sparse sources from multiple measurement vectors (MMV) under CS framework.

CS is an emerging area which can break through the limit of Nyquist sampling theorem. On one hand, CS can simultaneously capture and store compressed or sparse source at a rate much lower than that of Nyquist sampling. On the other hand, it can recover the original source using nonadaptive linear projection measurements onto a suitable measurement matrix which satisfies the RIP. In CS, the joint sparse recovery aims to find a common support shared by unknown sparse vectors from MMV, which is obtained by the measurement matrix. Support denotes the indices of the nonzero elements in the unknown sparse vectors. A sparse solution can be obtained as long as the support is determined.

CS theory has been widely applied to DOA estimation according to the source sparsity, which results from the fact that there are much fewer source directions than all potential directions in the spatial domain. The DOA estimation methods in CS are attractive since they have much better estimation performance than conventional estimation methods. In [15], Gürbüz et al. firstly formulate the DOA estimation problem under CS framework in the time domain. Wang et al. [16] propose a new architecture to estimate DOA by exploiting compressive sampling in the spatial domain. Stoica et al. [17] make full use of covariance matching criterion and present a semiparametric/sparse estimation method and its derivative called LIKES [18] for the separable model. Malioutov et al. [19] present the *l*_1_-SVD algorithm for DOA estimation which combines the SVD of the data matrix with a sparse recovery method based on *l*_1_-norm minimization. A new class of subspace-base algorithms such as compressive MUSIC (CS-MUSIC) [20] and subspace-augmented MUSIC (SA-MUSIC) [21] is proposed in recent years.

The RIP and various modified versions of it have been used as a foundation of performance guarantee [21–24] for the joint sparse recovery. The performance guarantee of MUSIC based on joint sparse recovery is given for identifying the unique support in a favorable case [25]. However, in the unfavorable case where the number of measurement vectors is smaller than the sparsity or the covariance matrix tends to lose rank due to correlated sources, performance guarantee fails. Lee et al. [21] propose a new performance guarantee in terms of a version of the RIP under such unfavorable conditions. The performance guarantees of other methods such as greedy algorithms and convex relaxation have been developed to find the sparse solution in [23, 24]. However, the guarantees of these methods cannot be simply extended to the MMV case to obtain a better bound for the sparse solution.

In this paper, we propose a novel augmented Lagrange based on modified covariance matching criterion method called AULMC for DOA estimation in CS. This method utilizes the minimization of the modified covariance matching criterion which is acquired by using regularization method to add penalties in order to obtain the stable sparse parameter estimation, especially in the low sparsity case. This minimization problem is shown to be a semidefinite program (SDP) and transformed into the constrained quadratic programming problem for the sake of reducing computational complexity. The augmented Lagrange function is formed to solve this problem by the use of augmented Lagrange method. AULMC has a number of advantages over the other methods. For example, it provides more precise estimation and higher resolution in the scenarios with low SNR, small number of snapshots, and closely spaced correlated sources, and it does not need priori knowledge about the number of sources or to choose the regularization parameter of the *l*_1_-optimization framework which is very difficult to select in the DOA estimation. In addition, we give a detailed derivation process of the closed-form expression for the Cramér-Rao bound (CRB) of the new method and discuss an explicit condition that guarantees performance of the new method. This performance guarantee is given in terms of weaker version of the RIP which is referred to as weak-1 RIP. Simulation results illustrate the performance of the proposed method.

It is worth noting that covariance matching criterion has been used for DOA estimation [26]. In [26], a sparse iterative covariance-base estimation method, abbreviated as SPICE, is proposed. Our approach is different from SPICE because it utilizes modified covariance matching criterion which can guarantee the stability of solution even if the source sparsity is rather low. In the future work, we will focus on the application of our approach to data-driven design [27–30]. Now we briefly summarize the contributions of this work as follows.

- A modified covariance matching criterion is proposed by adding penalties according to the regularization method.
- The original SDP problem is transformed into the constrained quadratic programming problem. The motivation to transform the problem is that it can reduce computational complexity.
- Augmented Lagrange based on modified covariance matching criterion method is devised to solve the resulting programming problem.
- The CRB and performance guarantee of the new method are given in detail.

The rest of this paper is organized as follows. In Section 2, we formulate the DOA estimation problem.  Section 3 presents a modified covariance matching criterion. A novel augmented Lagrange based on modified covariance matching criterion method for DOA estimation is described in detail in Section 4; the performance of which is analyzed in Section 5. The performance of the proposed method is evaluated by simulations in Section 6. Conclusions are provided in Section 7.

## 2. Problem Formulation

Consider *L* narrow-band sources ${\hat{s}}_{1},{\hat{s}}_{2},\ldots ,{\hat{s}}_{L}$ impinging on a sensor array that consisted of *P* omnidirectional sensors from far field. At time instant *t*, the array received source can be given by

(1) $$\begin{array}{c} x\left(t\right)=\overset{L}{\underset{k=1}{\sum }}a\left(\theta_{k}\right){\hat{s}}_{k}\left(t\right)+n\left(t\right), \end{array}$$

where **n**(*t*) ∈ *𝒞* ^*P*×1^ denotes a noise term, *θ*_*k*_ ∈ *Ω* is the unknown direction, of the *k*th source where *Ω* denotes the entire spatial range and **a**(*θ*_*k*_) is the *P* × 1 steering vector. Although the DOA estimation of the single snapshot, which is a typical single measurement vector (SMV) model, has its value, the number of snapshots is larger than one in the most practical applications. Correspondingly, the multiple snapshots model is a MMV model.

Let ${\left\{{\tilde{\theta }}_{k}\right\}}_{k=1}^{K}$ denote a fine grid which covers *Ω* where there exist *K*  (*K* ≫ *L*) potential directions of the sources *s*_1_, *s*_2_,…, *s*_*K*_ so that the true directions {*θ*_*k*_}_*k*=1_^*L*^ are aligned or are close to the grids. This means that if ${\tilde{\theta }}_{k_{1}},{\tilde{\theta }}_{k_{2}},\ldots ,{\tilde{\theta }}_{k_{L}}$ are equal to *θ*_1_, *θ*_2_,…, *θ*_*L*_, respectively, we have

(2) sk={s^l,  k=kl  (l=1,2,…,L),0,  elsewhere.

Hence, the multiple snapshots model can be written as the following sparse form:

(3) x(t)=∑k=1Ka(θ~k)sk(t)+n(t)=As(t)+n(t),  t=t1,t2…tM,

where $A=\left[a({\tilde{\theta }}_{1})\;\;\;\;a({\tilde{\theta }}_{2})\;\;\cdots \;\;a({\tilde{\theta }}_{K})\right]$ is the *P* × *K* manifold matrix corresponding to all the potential directions which is also referred to as an overcomplete dictionary in CS. $s(t)={\left[\begin{array}{cccc} s_{1}(t) & s_{2}(t) & \cdots & s_{K}(t) \end{array}\right]}^{T}$ is a *L*-sparse vector since it has at most *L* nonzero elements in *K* elements, and *L* is defined as sparsity where the operator [·]^*T*^ denotes transport. {**s**(*t*_*i*_)}_*i*=1_^*M*^ are jointly *L*-sparse if they share a common support. Hence, the matrix $S=\left[\begin{array}{cccc} s(t_{1}) & s(t_{2}) & \cdots & s(t_{M}) \end{array}\right]\in {𝒞}^{K\times M}$ has no more than *L* nonzero rows in order to be called row *L*-sparse. The MMV problem is that of identifying the row support of the unknown matrix **S** from the matrix **Y** ∈ *𝒞* ^*N*×*M*^ that consisted of MMV which is given by

(4) $$\begin{array}{c} Y=\left[y\left(t_{1}\right)\;\;\;\;y\left(t_{2}\right)\;\;\cdots \;\;y\left(t_{M}\right)\right]=𝚽AS+𝚽N \end{array}$$

with a common measurement matrix *𝚽* of the size *N* × *P* with *N* < *P* where *N* is the number of nonadaptive linear projection measurements, such as random Gaussian matrix or random partial Fourier matrix, and noise matrix **N**.

## 3. Modified Covariance Matching Criterion

In this section, the modified covariance matching criterion is developed in the CS scenario. A conventional covariance matrix of the compressed measurement source with the size *N* × *N* is given by

(5) $$\begin{array}{c} R_{y}=E\left[y\left(t\right)y^{*}\left(t\right)\right]=𝚽AR_{s}A^{*}{𝚽}^{*}+𝚽R_{n}{𝚽}^{*}, \end{array}$$

where **R**_*s*_ = *E*[**s**(*t*)s*(*t*)] is a *K* × *K* covariance matrix of the sparse source whose off-diagonal elements denote the source correlation and diagonal elements denote the source powers. Since the powers are one-to-one corresponding to all the potential directions and our focus is on the source angle parameter estimation, **R**_*s*_ can be reduced to a diagonal matrix $R_{s}(\tilde{\theta })=\mathrm{diag}\left(\begin{array}{cccc} {\tilde{\theta }}_{1} & {\tilde{\theta }}_{2} & \cdots & {\tilde{\theta }}_{K} \end{array}\right)$. According to (5), the measurement matrix can change the covariance matrix of the noise to render the noise colored even if the noise is white. Therefore, this adverse factor must be considered before recovering jointly sparse sources. In addition, the operators (·)* and *E*[·] denote conjugate transpose and expectation, respectively.

Since *𝚽* **R**_*n*_ 𝚽* is a positive definite Hermitian matrix, a prewhitened method is given by the Cholesky decomposition. Let **B** be the Cholesky factor that satisfies

(6) $$\begin{array}{c} {\left(𝚽R_{n}{𝚽}^{*}\right)}^{-1}=B^{*}B, \end{array}$$

where **B** ∈ *𝒞* ^*N*×*N*^ is an upper triangular matrix of positive line. Hence, a prewhitened process is implemented by multiplying **y**(*t*) by **B** in order to obtain a pure source whose covariance matrix is written as

(7) $$\begin{array}{c} {\tilde{R}}_{y}=B𝚽AR_{s}A^{*}{𝚽}^{*}B^{*}+I_{N}=CR_{s}C^{*}+I_{N}, \end{array}$$

where **C** = [**c**_1_, **c**_2_,…, **c**_*K*_] and **I**_*N*_ is an identity matrix of the size *N* × *N*. It is important to note that the unknown covariance matrix of the noise is transformed into the known identity matrix after the prewhitened process. Therefore, the prewhitened method improves the robustness to the noise. Then, the covariance matrix of the compressed measurement source denoising is realized by

(8) $$\begin{array}{c} R={\tilde{R}}_{y}-I_{N}=CR_{s}C^{*}. \end{array}$$

The parameter $\tilde{\theta }$ can be estimated by a class of the covariance matching estimation techniques (COMET) based on covariance matching criterion [31]. This parameter estimation method is well known to be a large-sample approximation of ML method and provide a more attractive solution than ML estimator.

The principle of COMET is that of using the right data to minimize its data model by the weighted least-squares (WLS) method. However, the lower the source sparsity is, the more likely it is to be ill-posed for the covariance matrix estimation error meaning that the optimal solution obtained directly by minimizing the conventional covariance matching criterion is instable. Hence, we employ regularization method to add penalties in order to sufficiently exploit prior knowledge to reduce the solution space for determining the stable optimal solution. The modified covariance matching criterion is proposed as the following form:

(9)   [vec⁡(R^)−vec⁡(R(θ~))]∗Γ∗P−1Γ[vec⁡(R^)−vec⁡(R(θ~))]  +αTr⁡[R−1(θ~)−R^−1]+βTr⁡[R−1(θ~)R^−14],

where parameter *α* ≥ 0 controls the solution smoothness (guarantee precision), parameter *β* ≥ 0 controls the solution scale (guarantee sparsity), the inverses of the matrix Γ, the sample covariance matrix $\hat{R}$, and $R(\tilde{\theta })$ are assumed to exist, and the matrix **P** ^−1^ is the inverse of the covariance matrix of the residuals, $\tilde{ɛ}=\Gamma \mathrm{vec}(\hat{R}-R(\tilde{\theta }))$. Since $\hat{R}$ is equal to $R(\tilde{\theta })$ as *M* → *∞*, it follows from [32] that $\mathrm{vec}(\hat{R}-R(\tilde{\theta }))$ satisfies the asymptotic normal distribution with mean zero and variance $N^{-1}R^{T}(\tilde{\theta })⊗\hat{R}$. In addition, the operators *Tr*⁡[·], *vec*⁡(·), and ⊗ denote matrix trace, column stacking operation, and Kronecker product, respectively. Then, the matrix **P** can be given by

(10) P=E[ɛ~(θ~)ɛ~∗(θ~)]=ΓE[vec⁡(R^−R(θ~))vec∗(R^−R(θ~))]Γ∗=ΓN−1RT(θ~)⊗R^Γ∗=ΓN−1DΓ∗.

We consider the normalized data model in (9) and choose the regularization parameters *α* = *β* = *N* based on perceptual criterion [33]. By substituting (10) into (9), we have

(11) vec∗(R^−R(θ~))D−1vec⁡(R^−R(θ~))  +Tr⁡[R−1(θ~)−R^−1]+Tr⁡[R−1(θ~)R^−1]4.

By the properties of *vec*⁡, ⊗, and *Tr*⁡, the data model can be further simplified to

(12) vec∗(R^−R(θ~))D−1vec⁡(R^−R(θ~))  +Tr⁡[R−1(θ~)(R^−R(θ~))R^−1]+Tr⁡[R−1(θ~)R^−1]4 =||R^−1/2(R^−R(θ~)+IN2)R−1/2(θ~)||2,

where ||·|| denotes the Frobenius norm for matrices or the Euclidean norm for vectors. The data model in (12) is considered to be the modified covariance matching criterion. It can be seen from (12) that a positive definite Hermitian matrix is added to the covariance matrix estimation error by applying penalties according to regularization method in order to guarantee the stability of solution.

## 4. DOA Estimation

In this section, we will utilize the minimization of the modified covariance matching criterion to estimate parameter $\tilde{\theta }$. Let

(13) $$\begin{array}{c} {\tilde{\theta }}_{s}=\arg\underset{\tilde{\theta }}{\min}{||{\hat{R}}^{-1/2}\left(\hat{R}-R\left(\tilde{\theta }\right)+\frac{I}{2}\right)R^{-1/2}\left(\tilde{\theta }\right)||}^{2} \end{array}$$

be the optimal solution of  $\tilde{\theta }$ in the structure model of (8). Our goal is to utilize the modified covariance matching criterion for an estimate that is asymptotically equal to ${\tilde{\theta }}_{s}$. By the properties of the trace and the Hermitian matrix, a derivation process is shown as follows, where we omit the dependence on  $\tilde{\theta }$ for notational convenience:

(14) f=Tr⁡[R^−1(R^−R+I2)R−1(R^−R+I2)]=Tr⁡[R^−1R−R^−1+(R^1/2+12R^−1/2)·R−1/2(R^1/2+12R^−1/2)R−1/2].

Due to

(15) $$\begin{array}{c} \mathrm{Tr}\left[{\hat{R}}^{-1}R\right]=\overset{K}{\underset{k=1}{\sum }}\left(c_{k}^{*}{\hat{R}}^{-1}c_{k}\right){\tilde{\theta }}_{k} \end{array}$$

we can deduce that the minimization of *f* is equal to the minimization of *h*:

(16) h=Tr⁡[(R^1/2+12R^−1/2)R−1/2(R^1/2+12R^−1/2)R−1/2]+∑k=1K(ck∗R^−1ck)θ~k.

Then, we will demonstrate that the minimization of *h* in (16) with respect to ${\left\{{\tilde{\theta }}_{k}\right\}}_{k=1}^{K}$ is an SDP. To prove this fact, One assumes

(17) $$\begin{array}{c} {\hat{R}}^{1/2}+\frac{1}{2}{\hat{R}}^{-1/2}=\left[\begin{array}{cccc} r_{1} & r_{2} & \cdots & r_{N} \end{array}\right]. \end{array}$$

The *h* in (16) can be rewritten as

(18) $$\begin{array}{c} h=\overset{N}{\underset{k=1}{\sum }}r_{k}^{*}R^{-1}r_{k}+\overset{K}{\underset{k=1}{\sum }}\left(c_{k}^{*}{\hat{R}}^{-1}c_{k}\right){\tilde{\theta }}_{k}. \end{array}$$

By the Schur complement, let {*x*_*k*_}_*k*=1_^*N*^ be auxiliary variables satisfying

(19) [xkrk∗rkR]≥0.

The equivalent form of this minimization problem is expressed as

(20)  min⁡xk,    θ~k ∑k=1Nxk+∑k=1K(ck∗R^−1ck)θ~k s.t.  θ~k≥0, k=1,2,…,K,    [xkrk∗rkR]≥0, k=1,2,…,N.

It is easy to see that (20) is an SDP [34]. Many software packages can solve an SDP, but solving (16) as an SDP is not a good choice because SDP solvers have generally rather high computational complexity for the values of *N*, *M*, and *K* in the DOA estimation. To solve it effectively, we transform it into the constrained quadratic programming problem for reducing computational complexity, as described next.

Since a consistent estimation of $\tilde{\theta }$ is given by (15), we can reformulate the minimization of *h* in (16) as the following constrained minimization by the Schur inequality of the trace:

(21) min⁡θi≥0Tr⁡[(R^1/2+12R^−1/2)R−1/2(R^1/2+12R^−1/2)R−1/2] s.t. Wz=1K,

where **W** is a *K* × *K* diagonal matrix of ${\left[C^{*}{\hat{R}}^{-1}C\right]}^{-1/2}$ and $z={\left[\begin{array}{cccc} z_{1} & z_{2} & \cdots & z_{K} \end{array}\right]}^{T}$ is a *K*-dimensional vector with *z*_*i*_ = *θ*_*i*_^−1/2^, *i* = 1,2,…, *K*. By substituting (8) into (21), the objective function in (21) can be rewritten as

(22) Tr⁡[(R^1/2+12R^−1/2)(C∗)−1/2Rs−1/2C−1/2·(R^1/2+12R^−1/2)(C∗)−1/2Rs−1/2C−1/2] =Tr⁡[C−1/2(R^1/2+12R^−1/2)(C∗)−1/2Rs−1/2·C−1/2(R^1/2+12R^−1/2)(C∗)−1/2Rs−1/2].

Based on the following equation:

(23) $$\begin{array}{c} \mathrm{Tr}\left[MT\left(d\right)NT\left(d\right)\right]=d^{*}\left(M^{T}⊙N\right)d, \end{array}$$

where ⊙ denotes the Schur-Hadamard product, **M** and **N** are both *K* × *K* matrices, **T**(**d**) = diag⁡(*d*_1_ *d*_2_   ⋯   *d*_*K*_), and $d={\left[\begin{array}{cccc} d_{1} & d_{2} & \cdots & d_{K} \end{array}\right]}^{T}$, the objective function (22) can be written as

(24) $$\begin{array}{c} \mathrm{Tr}\left[{QR}_{s}^{-1/2}{QR}_{s}^{-1/2}\right]=z^{*}Vz, \end{array}$$

where $Q=C^{-1/2}({\hat{R}}^{1/2}+(1/2){\hat{R}}^{-1/2}){\left(C^{*}\right)}^{-1/2}$ and **V** = **Q** ^*T*^⊙**Q**. Hence, the minimization problem in (21) is transformed into the following form:

(25) min⁡z  f(z)=z∗Vz s.t. Π(z)=Wz−1K=0K

which is a typical constrained quadratic programming problem.

It is well known that an important class of methods for solving the constrained quadratic programming problem is to form the auxiliary function. To solve (25), we form the following augmented Lagrange function with respect to (25):

(26) $$\begin{array}{c} p_{\sigma }\left(z,\bar{\mu }\right)=f\left(z\right)+{\bar{\mu }}^{*}\Pi \left(z\right)+\frac{1}{2}\sigma {||\Pi \left(z\right)||}^{2}, \end{array}$$

where $\bar{\mu }$ is the asymptotical solution of the Lagrange multiplier of (25) and *σ* is a penalty factor.

By setting the gradient and the Hessian matrix of (26) with respect to **z** to zero, we have

(27) ∇zpσ(z,μ¯)=∇zl(z,μ¯)+σq(z)Π(z),∇zz2pσ(z,μ¯)=∇zz2l(z,μ¯)+σ∑i=1KΠi(z)∇2Π(z)+σq(z)q∗(z),

where $l(z,\bar{\mu })=f(z)+{\bar{\mu }}^{*}\Pi (z)$ and $q(z)=\nabla \Pi^{*}(z)=\left[\begin{array}{cccc} \nabla \Pi_{1}(z) & \nabla \Pi_{2}(z) & \cdots & \nabla \Pi_{K}(z) \end{array}\right]$. One assumes that

(28) $$\begin{array}{c} b\left(z,\bar{\mu }\right)=\nabla_{zz}^{2}l\left(z,\bar{\mu }\right)+\sigma \overset{K}{\underset{i=1}{\sum }}\Pi_{i}\left(z\right)\nabla^{2}\Pi \left(z\right). \end{array}$$

Applying the Newton method, we obtain

(29) $$\begin{array}{c} \nabla_{zz}^{2}p_{\sigma_{k}}\left(z_{k},{\bar{\mu }}_{k}\right)\left(z_{k+1}-z_{k}\right)=-\nabla_{z}p_{\sigma_{k}}\left(z_{k},{\bar{\mu }}_{k}\right). \end{array}$$

For notational convenience, we assume that **q**_*k*_ = **q**(**z**_*k*_), ∇*f*_*k*_ = ∇*f*(**z**_*k*_), Π_*k*_ = Π(**z**_*k*_), $b_{k}=b(z_{k},{\bar{\mu }}_{k})$, **d**_*k*_ = **z**_*k*+1_ − **z**_*k*_ and we get the following equation by (29):

(30) $$\begin{array}{c} b_{k}d_{k}+\sigma_{k}q_{k}q_{k}^{*}d_{k}+\nabla_{z}l\left(z_{k},{\bar{\mu }}_{k}\right)+\sigma_{k}q_{k}\Pi_{k}=0. \end{array}$$

Assuming that the inverse of  **b**_*k*_ exists, by left-multiplying (30) by (1/*σ*_*k*_)**q**_k_*b_*k*_^−1^, we have

(31) (IKσk+qk∗bk−1qk)qk∗dk  =−qk∗bk−1qkΠk−1σkqk∗bk−1∇zl(zk,μ¯k).

It follows from (31) that

(32) $$\begin{array}{c} \;\;\;\;q_{k}^{*}b_{k}=-\Pi_{k}+\frac{\alpha_{k}}{\sigma_{k}}, \end{array}$$

where **α**_*k*_ satisfies

(33) $$\begin{array}{c} \left(\frac{I_{K}}{\sigma_{k}}+q_{k}^{*}b_{k}^{-1}q_{k}\right)\alpha_{k}=-q_{k}^{*}b_{k}^{-1}\nabla_{z}l\left(z_{k},{\bar{\mu }}_{k}\right)+\Pi_{k}. \end{array}$$

By substituting (32) into (30), we have

(34) $$\begin{array}{c} d_{k}=-b_{k}^{-1}\left(q_{k}\alpha_{k}+\nabla_{z}l\left(z_{k},{\bar{\mu }}_{k}\right)\right). \end{array}$$

Both the multiplier factor and the penalty factor are determined with difficulty for utilizing the augmented Lagrange function to solve the constrained quadratic programming problem. Hence, an updated sequence for the multiplier factor is given in terms of Proposition 1.

**Proposition 1**

One assumes that ***μ***(**z**) is the optimal solution of the problem min⁡_***μ***∈*𝒞*^*K*^_||**q**(**z**)***μ***+∇*f*(**z**)||^2^. Then, the following equation holds:

(35) $$\begin{array}{c} \mu \left(z_{k}\right)={\bar{\mu }}_{k}+\alpha_{k}+\beta_{k}d_{k}, \end{array}$$

where **β**_*k*_ = (**q**_k_*q_*k*_)^−1^ **q**_k_*b_*k*_.

**Proof**

See Appendix A.

It can be deduced from Proposition 1 that ${\bar{\mu }}_{k}+\alpha_{k}$ is referred to as the next iteration of ${\bar{\mu }}_{k}$. We apply a heuristic update sequence for the penalty factor to achieve a stable solution. If the *k*th iterative solution **z**_*k*_ is closer to the feasible region than the previous solution **z**_*k*−1_, the penalty factor is decreased. Inversely, we increase the penalty factor when **z**_*k*_ is not closer to the feasible region.

The specific steps of solving by the augmented Lagrange method are given as follows.

Initialization: set *k* = 1, **z**_1_ ∈ *𝒞* ^*K*^, *σ*_1_ > 0, *τ* ∈ (0,1), and ${\bar{\mu }}_{1}=-{\left(q_{1}^{*}q_{1}\right)}^{-1}q_{1}^{*}\nabla f_{1}$.

(1) Calculate **α**_*k*_ and **d**_*k*_ in terms of (33) and (34). If ||**d**_*k*_||≃0, **z**_*k*_ is the KKT point of the problem (25); then stop iteration.
(2) We use Armijo search method to find the maximum of *t*_*k*_ satisfying
  (36) $$\begin{array}{c} p_{\sigma_{k}}\left(z_{k}+t_{k}d_{k},{\bar{\mu }}_{k}\right)\leq p_{\sigma_{k}}\left(z_{k},{\bar{\mu }}_{k}\right)+\tau t_{k}\nabla p_{\sigma_{k}}^{*}\left(z_{k},{\bar{\mu }}_{k}\right)d_{k}. \end{array}$$
(3) Set **z**_*k*+1_ = **z**_*k*_ + *t*_*k*_ **d**_*k*_ and update the multiplier factor and penalty factor, respectively:
  (37) μ¯k+1=μ¯k+αk,σk+1={2σkif  ||Π(zk+1)||>||Π(zk)||,12σkif  ||Π(zk+1)||≤||Π(zk)||.
(4) Set *k* = *k* + 1 and return to step (1).

## 5. Performance Analysis

### 5.1. Cramér-Rao Bound

In this subsection, the closed-form expression for the CRB of the proposed method with complex white Gaussian noise after the prewhitened process is illustrated. The bound of the noise variance estimation can be computed separately as CRB_*n*_ = 1/*NM* (see [35]). The remaining parameters consist of an unknown vector $\varphi ={\left[\begin{array}{cc} {\tilde{\theta }}^{T} & s^{T} \end{array}\right]}^{T}$. It is not an easy task to get the CRB of the unknown parameters. However, fortunately, [36, 37] have provided a critical inspiration for the derivation in this paper.

First, the likelihood function is given by

(38) L(ys(tj),θ~)=1(2π)MN(1/2)MN·exp⁡{−∑j=1M(y(tj)−B𝚽As(tj))∗×(y(tj)−B𝚽As(tj))}.

Thus, the log-likelihood function is

(39) ln⁡L(ys(tj),θ~)=const−∑j=1M(y(tj)−B𝚽As(tj))∗×(y(tj)−B𝚽As(tj)).

Then, the partial derivatives of (39) with respect to $\tilde{\theta }$, **s**_*r*_(*t*_*j*_) = *Re*{**s**(*t*_*j*_)} and **s**_*i*_(*t*_*j*_) = *Im*⁡{**s**(*t*_*j*_)} are given by

(40) ∂ln⁡L∂θ~=2 ∑j=1MRe{Sj∗U∗𝚽∗B∗n(tj)},∂ln⁡L∂sr(tj)=2 Re{A∗𝚽∗B∗n(tj)},∂ln⁡L∂si(tj)=2 Im⁡{A∗𝚽∗B∗n(tj)},

where **S**_*j*_ = diag⁡(**s**(*t*_*j*_)) and $U=\left[\begin{array}{cccc} da({\tilde{\theta }}_{1})/d{\tilde{\theta }}_{1} & da({\tilde{\theta }}_{2})/d{\tilde{\theta }}_{2} & \cdots & da({\tilde{\theta }}_{K})/d{\tilde{\theta }}_{K} \end{array}\right]$. Following [35, 37], we can obtain the Fisher information matrix (FIM) as follows:

(41) FIM=Λ−[Δ1r    Δ1i    Δ2r        ⋯    ΔMi    ΔMr]·[Gr−Gi0⋯0GiGr⋱⋮0⋱0⋮⋱Gr−Gi0⋯0GiGr]·[Δ1rΔ1iΔ2r⋮ΔMrΔMi],

where

(42) E{∂ln⁡L∂θ~(∂ln⁡L∂θ~)T}=Λ=2∑j=1MRe{Sj∗U∗𝚽∗B∗B𝚽USj},E{∂ln⁡L∂sr(tk)(∂ln⁡L∂si(tp))T}=−Gi−1=−Im⁡{G−1}=−Im⁡{2A∗𝚽∗B∗B𝚽A}δkp,E{∂ln⁡L∂sr(tk)(∂ln⁡L∂sr(tp))T}=E{∂ln⁡L∂si(tk)(∂ln⁡L∂si(tp))T}=Gr−1=Re{G−1}=Re{2A∗𝚽∗B∗B𝚽A}δkp,E{∂ln⁡L∂sr(tj)(∂ln⁡L∂θ~)T}=Re{Δj}=Δjr=Re{2A∗𝚽∗B∗B𝚽USj},     j=1,2,…,M,E{∂ln⁡L∂si(tj)(∂ln⁡L∂θ~)T}=Im⁡{Δj}=Δji=Im⁡{2A∗𝚽∗B∗B𝚽USj},     j=1,2,…,M.

It is well known that

(43) [ΔrTΔiT][Gr−GiGiGr][ΔrΔi]=Re{Δ∗GΔ}.

It can be deduced from (41) and (43) that

(44) CRB(θ~)=FIM−1=12{∑j=1MRe{Sj∗U∗H∗(I−HA(HA)+)HUSj}}−1,

where **H** = **B** *𝚽* is a *N* × *P* matrix and (·)^+^ denotes pseudoinverse. Note that the CRB in CS is affected not only by the conventional factors, for example, SNR, array structure and signal relative location, but also by the measurement matrix.

### 5.2. Performance Guarantee

In CS, the RIP has been deeply studied for the joint sparse recovery by minimizing the *l*_1_ norm. We say that the matrix **C** ∈ *𝒞* ^*N*×*K*^ obeys the RIP of the order *L* if there exists a constant *δ* ∈ (0,1) satisfying

(45) $$\begin{array}{c} \left(1-\delta \right){||s||}_{2}^{2}\ll {||Cs||}_{2}^{2}\ll \left(1+\delta \right){||s||}_{2}^{2}. \end{array}$$

Therefore, all submatrices of **C** with **L** columns are uniformly well conditioned. The restricted isometry constant (RIC) of the order **L**, described as *δ*_*L*_(**C**), is the smallest *δ* that satisfies (45) and *δ*_*L*_(**C**) satisfies

(46) $$\begin{array}{c} \delta_{L}\left(C\right)=\underset{\left|J^{\prime }\right|=L}{\max}||C_{J^{\prime }}^{*}C_{J^{\prime }}-I_{L}||, \end{array}$$

where *J*′ is a *L*-dimensional subsupport of *J* = [1,2,…, *K*] and **C**_*J*′_ denotes the submatrix of **C** with columns indexed by *J*′. Note that the condition satisfying the RIP is so demanding that its applications are limited. Therefore, we should make use of a new version of the RIP, which is called the weak-1 RIP [38], to control the size of the recovery error. The weak-1 RIP is given in the following form:

(47) $$\begin{array}{c} \left(1-\alpha \right){||v||}_{2}^{2}\ll {||Cv||}_{2}^{2}\ll \left(1+\alpha \right){||v||}_{2}^{2} \end{array}$$

for all **v** supported on *U*, where the cardinality of the set *U* is *L* + 1. If the matrix **C** satisfies the weak-1 RIP, it can be deduced that 0 ≤ *α* ≤ *ϕ*_*L*+1_(**C**_*U*_), where *ϕ*_*k*_(**C**_*U*_) denotes the *k*th largest eigenvalue of **C**_*U*_. The corresponding weak-1 RIC is given by

(48) $$\begin{array}{c} \alpha_{L+1}^{w}\left(C;U\right)=\min\phi_{L+1}\left(C_{U}\right). \end{array}$$

In this paper, when the estimation quality is imperfect, especially in the unfavorable case, the support is no longer identified by the algebraic property of **R**. Hence, a new performance guarantee is given in terms of the weak-1 RIP in the following proposition.

**Proposition 2**

One assumes that $F={\hat{R}}^{1/2}+(1/2){\hat{R}}^{-1/2}$ and $R^{-1}=𝚿R_{s}^{-1}(\tilde{\theta }){𝚿}^{*}$ where $𝚿=\left[\begin{array}{cccc} \psi_{1} & \psi_{2} & \ldots & \psi_{K} \end{array}\right]$. $\hat{\theta }$ is an estimation that is asymptotically equal to ${\tilde{\theta }}_{s}$ such that $|\mathrm{tr}(R^{-1}(\hat{\theta }))-\mathrm{tr}(R^{-1}({\tilde{\theta }}_{s}))|\leq \eta$ for *η* ∈ (0, 1/2||**F**||_2_^2^). Let *J*_0_ and *J*_1_ = {*j*_*i*_}_*i*=1_^*L*^ be the *L*-dimensional supports that consisted of the indexes of *L* largest elements in ${\tilde{\theta }}_{s}$ and $\hat{\theta }$, respectively. If the matrix **R** satisfies

(49) $$\begin{array}{c} \alpha_{L+1}^{w}\left(R;J_{0}\right)>\kappa \end{array}$$

for

(50) $$\begin{array}{c} \kappa \geq \frac{L{||F||}_{2}^{2}\sum_{j\in J_{0}}\psi_{j}^{*}\psi_{j}}{1-2\eta {||F||}_{2}^{2}} \end{array}$$

the support can be identified.

**Proof**

See Appendix B.

It follows from Proposition 2 that the performance guarantee of the proposed method requires a mild condition in the unfavorable case. However, in the favorable case, the performance guarantee only requires a much milder condition, *α*_*L*+1_^*w*^(**R**; *J*_0_) > 0, which is an algebraic condition.

## 6. Simulation Results

In this section, the performance of the proposed method is illustrated by several simulation results and compared with that of CS-MUISC, SPICE, and CRB under the condition that the number of sources is unknown. We consider the spatial signal impinging on the uniform linear array (ULA) with interspacing *λ*/2 where *λ* denotes the wavelength of source. In the ULA case, the steering vector corresponding to the DOA equal to *θ*_*k*_ is given by

(51) a(θk)=[1e−jπsin(θk)⋯e−j(P−1)πsin(θk)]T,

where the number of the array elements is set to be *P* = 8.

In the simulation, the average root mean square error (RMSE) of the DOA estimation with 50 Monte Carlo runs is defined as the significant performance index:

(52) $$\begin{array}{c} \text{RMSE}={\left[\overset{50}{\underset{m=1}{\sum }}\;\overset{L}{\underset{l=1}{\sum }}\frac{{\left({\bar{\theta }}_{lm}-\theta_{l}\right)}^{2}}{50L}\right]}^{1/2}, \end{array}$$

where ${\bar{\theta }}_{lm}$ is the estimate of *θ*_*l*_ in the *m*th run.

The resolution of the grid is closely related to the precision of the DOA estimation. A coarse grid can lead to poor precision, but a too fine grid increases computational complexity. Therefore, an adaptive grid refinement method is used to balance the tradeoff between precision and computational complexity. In the simulation, we make a coarse grid with 1° step in the range of −90° to 90° and perform a local fine grid in the vicinity of locations obtained by using the coarse grid.

**Figure 1:**
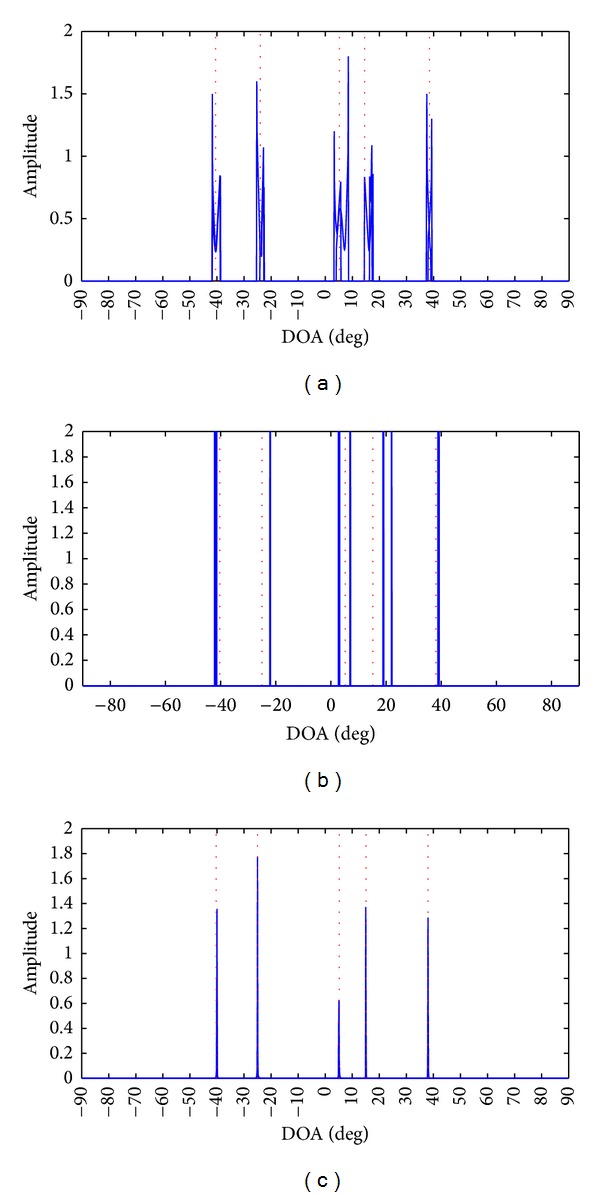
Superimposed spatial spectra of CS-MUSIC, SPICE, and AULMC in 10 Monte Carlo runs, where the red vertical dashed lines denote the true DOAs. (a) CS-MUSIC, (b) SPICE and (c) AULMC.

In the first simulation, we display the superimposed spatial spectra of three algorithms in 10 Monte Carlo runs in the scenario with low SNR, small number of snapshots, and five sources impinging from $\left[\begin{array}{ccccc} -40.3^{{}^{\circ}} & -25^{{}^{\circ}} & 5.2^{{}^{\circ}} & 15.2^{{}^{\circ}} & 38.1^{{}^{\circ}} \end{array}\right]$, respectively where two most closely spaced sources are correlated and the remaining sources are uncorrelated. The spatial spectra are shown in Figure 1 with 3 dB SNR and 50 snapshots. The following facts can be acquired from Figure 1 as follows: the spatial spectrum obtained by CS-MUSIC suffers from severe interference at the true directions, especially at two most closely spaced correlated sources whose bias is clearly seen from the insert. Two most closely spaced correlated sources can be resolved by SPICE (note that peaks in the spatial spectrum are much larger than 2, but they are cut off at 2 to use the same scale as the other figures in Figure 1), but SPICE can yield false peaks and slight bias in the vicinity of the correlated sources and uncorrelated sources, respectively. The proposed method AULMC yields a nearly ideal spatial spectrum and provides a precise estimation for all the sources. In summary, AULMC outperforms CS-MUSIC and SPICE in terms of the spatial spectrum in the scenario with low SNR, small number of snapshots, and closely spaced correlated sources.

**Figure 2:**
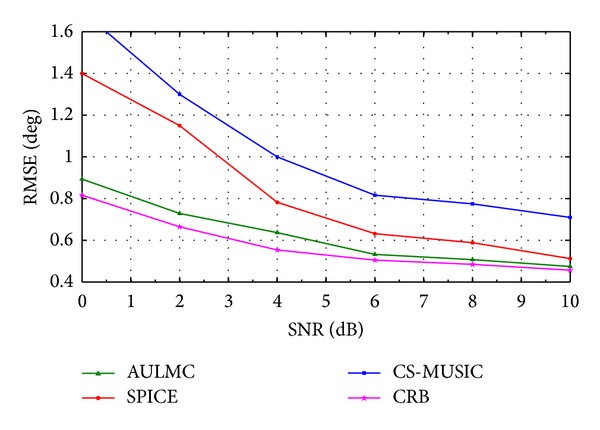
RMSE of the DOA estimation versus SNR for 50 snapshots.

**Figure 3:**
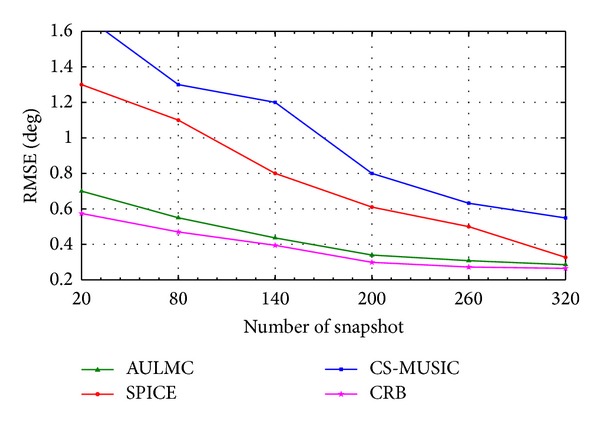
RMSE of the DOA estimation versus number of snapshots for 5 dB SNR.

We analyze the RMSE of three algorithms under different conditions in the second simulation. The source model is the same as the first simulation.  Figure 2 shows the RMSE as a function of SNR of all the algorithms and CRB in 50 Monte Carlo runs for the fixed number of snapshots 50, whereas the RMSE versus number of snapshots is shown in Figure 3 for the fixed SNR 5 dB in 50 Monte Carlo runs. Based on Figures 2 and 3, we can draw the conclusions that the RMSE of AULMC is smaller than those of other two algorithms and AULMC has the more significant performance advantages than the other two algorithms, especially in the scenarios with low SNR or small number of snapshots. One possible explanation is that AULMC can give the stable estimation in every Monte Carlo run. It can be also seen that the RMSE is close to the CRB with the increase of SNR and the number of snapshots.

**Figure 4:**
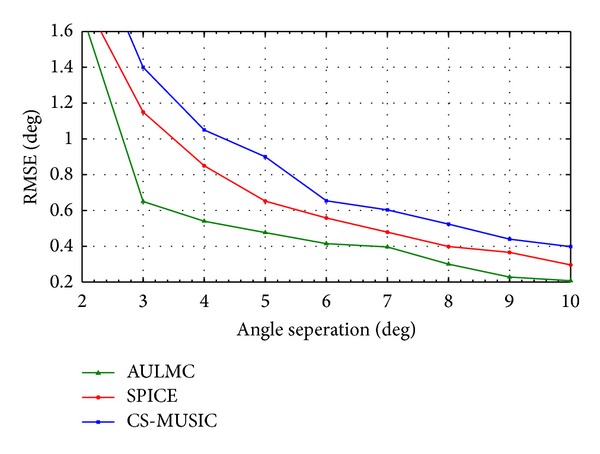
RMSE of the DOA estimation versus angle separation, where the SNR is 13 dB and the number of snapshots is 100.

In Figure 4, we display the relation between the RMSE and angel separation of correlated sources which can illustrate the resolving ability. Let two correlated sources at angles 20° and 20° + Δ*θ*, where the step of Δ*θ* is 1°, be impinged on the ULA. The SNR is 13 dB and the number of snapshots is 100. It can be seen from Figure 4 that when angle separation is 2°, AULMC fails; however, AULMC can still provide a precise estimation as long as the angle separation is no less than 3° and has higher resolution than the other two algorithms.

Finally, the computation time of different algorithms versus number of snapshots is shown in Table 1 for comparing the efficiency of these algorithms and SDP solver. Two correlated sources impinge on the ULA at 20° and 25°. The SNR is fixed at 13 dB. The computation time is obtained by the MATLAB 7.8 (R2009a) on a 2.8 GHz 4 GB PC. For AULMC, the computation time is mainly spent on the iterations of augmented Lagrange.

It can be seen from Table 1 that the computation time of SDP solver is the longest, and although the computation time of AULMC is longer than that of other two algorithms, it is comparable. Moreover, it is worth noting that the performance of AULMC is much better than that of CS-MUSIC or SPICE.

## 7. Conclusion

A novel augmented Lagrange based on modified covariance matching criterion method for DOA estimation is proposed in CS. It is proved that the problem of minimizing the modified covariance matching criterion is an SDP, which can be transformed into the constrained quadratic programming problem solved by the augmented Lagrange method. A detailed derivation for the CRB and a theoretical performance guarantee for identifying the support are provided. Simulation results show that AULMC outperforms CS-MUSIC and SPICE in terms of the spatial spectrum and has more precise estimation as well as higher resolution, especially in the scenarios with low SNR, small number of snapshots, and closely spaced correlated sources.

## Figures and Tables

**Table 1:** Comparison of computation time.

Number of snapshots	Time (sec)			
	AULMC	CS-MUSIC	SPICE	SDP solver
50	0.0651	0.0569	0.0601	0.7526
75	0.0776	0.0604	0.0714	0.8871
100	0.0860	0.0640	0.0819	1.0268
125	0.0974	0.0691	0.0923	1.3018

## Appendices

### A. Proof of Proposition 1

Due to ***μ***(**z**_*k*_) = −(**q**_*k*_***q**_*k*_)^−1^ **q**_*k*_*∇*f*_*k*_, we have

(A.1) $$\begin{array}{c} q_{k}^{*}q_{k}\mu \left(z_{k}\right)=-q_{k}^{*}\nabla f_{k}. \end{array}$$

With (34) and (A.1), we get

(A.2) $$\begin{array}{c} q_{k}^{*}q_{k}\left[\mu \left(z_{k}\right)-{\bar{\mu }}_{k}-\alpha_{k}\right]=q_{k}^{*}b_{k}d_{k}. \end{array}$$

Therefore, it is implied by (A.2) that (35) holds and the proof of Proposition 1 is completed.

### B. Proof of Proposition 2

The support *J*_0_ can be identified if

(B.1) max⁡j∈J0Tr⁡[FR−1/2(θ^)FR−1/2(θ^)]Tr⁡[F2]   <min⁡j∈J∖J0Tr⁡[FR−1/2(θ^)FR−1/2(θ^)]Tr⁡[F2].

By the Cauchy-Schwarz inequality of the trace, we have

(B.2) |Tr⁡[(FR−1/2(θ^))2]−Tr⁡[(FR−1/2(θ~s))2]Tr⁡[F2]|   ≤Tr⁡[F2]·|Tr⁡[R−1(θ^)]−Tr⁡[R−1(θ~s)]|Tr⁡[F2]   =|Tr⁡[R−1(θ^)]−Tr⁡[R−1(θ~s)]|≤η.

Then, for all *j* ∈ *J*_0_ we have

(B.3) Tr⁡[(FR−1/2(θ^))2]Tr⁡[F2]≤η+Tr⁡[(FR−1/2(θ~s))2]Tr⁡[F2]≤η+Tr⁡[F2]Tr⁡[R−1(θ~s)]Tr⁡[F2]=η+∑j∈J0ψj∗ψjθ~j≤η+L∑j∈J0ψj∗ψjαL+1w(R;J0).

Thus, an upper bound on the left-band side of (B.1) is given by

(B.4) $$\begin{array}{c} \underset{j\in J_{0}}{\max}\frac{\mathrm{Tr}\left[{\left(FR^{-1/2}\left(\hat{\theta }\right)\right)}^{2}\right]}{\mathrm{Tr}\left[F^{2}\right]}\leq \eta +\frac{L\sum_{j\in J_{0}}\psi_{j}^{*}\psi_{j}}{\alpha_{L+1}^{w}\left(R;J_{0}\right)}. \end{array}$$

Similarly, we can obtain

(B.5) Tr⁡[(FR−1/2(θ^))2]Tr⁡[F2]≥Tr⁡[F2R−1(θ~s)]Tr⁡[F2]−η=∑j∈J∖J0ψj∗F2ψj/θ~jTr⁡[F2]−η≥1||F||22−η

for all *j* ∈ *J*∖*J*_0_ and hence describe a lower bound on the right-hand side of (B.1) as

(B.6) $$\begin{array}{c} \underset{j\in J\setminus J_{0}}{\min}\frac{\mathrm{Tr}\left[{\left(FR^{-1/2}\left(\hat{\theta }\right)\right)}^{2}\right]}{\mathrm{Tr}\left[F^{2}\right]}\geq \frac{1}{{||F||}_{2}^{2}}-\eta . \end{array}$$

Combining (B.4) with (B.6), the support *J*_0_ can be identified if **R** satisfies

(B.7) $$\begin{array}{c} \alpha_{L+1}^{w}\left(R;J_{0}\right)>\kappa \end{array}$$

for

(B.8) $$\begin{array}{c} \kappa \geq \frac{L{||F||}_{2}^{2}\sum_{j\in J_{0}}\psi_{j}^{*}\psi_{j}}{1-2\eta {||F||}_{2}^{2}}. \end{array}$$
